# The effect of an extra piece of fruit or vegetables at school on weight status in two generations - 14 years follow-up of the Fruit and Vegetables Makes the Marks study

**DOI:** 10.1371/journal.pone.0205498

**Published:** 2018-10-15

**Authors:** Tonje Holte Stea, Eline Tønnesson Tveter, Saskia J. te Velde, Frøydis Nordgård Vik, Knut-Inge Klepp, Elling Bere

**Affiliations:** 1 University of Agder, Department of Public Health, Sport and Nutrition, Kristiansand, Norway; 2 University of Oslo, Department of Nutrition, Oslo, Norway; Vanderbilt University, UNITED STATES

## Abstract

**Background:**

The obesity epidemic presents a major public health challenge, and a poor diet quality has been identified as one of the most important contributing factors. Whereas a sufficient fruit and vegetable consumption has been associated with several positive health outcomes, the long-term effect on overweight and obesity is unclear. Thus, the aims of this study were to investigate if one year with free school fruit had any effect on weight status 14 years later, and if it affected the birth weight of the participants’ children.

**Methods:**

In 2001, 10 -12-year old Norwegian children, received one year of free school fruit in the intervention study “Fruits and Vegetables Make the Marks” (FVMM) and in 2016, a total of 1081 participants of 2049 eligible responded to a follow-up survey. Multilevel logistic regression was used to investigate if one year of free school fruit was associated with weight status and with birthweight status of the offspring. The analyses were adjusted for gender, educational level, and the offspring analysis also for parents’ weight status, and the nested design (child/parent).

**Results:**

The odds ratios of being overweight (OR: 0.93, 95% CI: 0.70, 1.24) or having a child with high or low birth weight (OR: 0.52, 95% CI: 0.21, 1.30) in the intervention group compared to the control group were not statistically significant, 14 years after the intervention period.

**Conclusions:**

One year of free school fruit did not have an effect on weight status on the participants or birth weight of their offspring, 14 years after the intervention period. Although, results from the present study contribute to fill the knowledge gaps concerning long-term effects of public health efforts on weight status, more follow-up studies with larger samples are warranted.

## Introduction

Overweight and obesity are causing challenging public health problems worldwide, and in most countries, rates of overweight and obesity are continuing to rise [1–3]. Overweight and obesity are associated with several adult chronic illnesses, such as coronary heart disease, type 2 diabetes, and some types of cancer [3,4]. Studies have also shown a strong dose-response association between pre-pregnancy BMI, offsprings`birth weight and childhood BMI [5–8], and childhood overweight and obesity has shown to increase the risk of adult obesity and chronic disease [3]. Weight-control interventions among overweight and obese pregnant women have shown limited effects on birth weight of the child [9–11], and it has been concluded that lifestyle changes before pregnancy seem more promising than during pregnancy [11].

Unhealthy dietary habits are closely related to risk of overweight and obesity [3]. Intake of fruit and vegetables (FV), which are low in energy and rich in fibre, has shown to reduce the risk of adiposity [12] and has also been linked to reduced risks for cardiovascular diseases and several cancers [13–16]. Therefore, the importance of promoting healthy lifestyle habits, including increased consumption of FV, from an early age using effective surveillance, prevention, and treatment programs has been underlined [17,18].

Systematic review studies have reported that school-based intervention programmes tend to be effective in increasing pupils’ FV intake [19,20], and that school based intervention programmes may effectively contribute in preventing overweight and obesity [21]. Although most of the previously published school-based intervention studies have only reported short-term effects on weight status, few studies have investigated long-term effects [20,21]. Two systematic review studies reported that only eight out of 48 included studies investigated follow-up effects more than a year after the intervention period [19,20] and only one of the reviewed studies, a two-year follow-up study, showed a positive impact on FV consumption and BMI [19]. In addition, the Fruit and Vegetables Make the Marks (FVMM) study, in which Norwegian school-children received one piece of fruit or vegetables for free for one school year (2001/2002), reported a lower prevalence of overweight in the intervention group compared to the control group (15% vs. 25% overweight) seven years after the FVMM intervention period [22]. However, the association was not significant in the final model, and the validity of this finding was hampered by several limitations, including a very low participation rate.

Due to limited scientific evidence from school-based intervention studies and other studies, and a limited number of long-term longitudinal follow up studies, we are not able to conclude that FV consumption has a protective effect on the risk of developing obesity [19,20,23]. Thus, long-term follow-up of school-based intervention studies investigating the association between dietary patterns and weight status during transition from childhood to adolescence, and further into adulthood has been warranted [24,25]. Our study aims to fill this knowledge gap by evaluating the effect of the 15 years follow-up of providing free school fruit for one year in elementary schools on (1) the weight status of the participants and (2) the birth weight of their children.

## Methods

### Study design

In 2001, 48 schools from Hedmark and Telemark counties (24 schools in each county) were randomly selected and invited to participate in the research project FVMM. Nineteen schools from each county agreed to participate [26]. Nine schools within one of the counties were randomly selected as intervention schools and participated in the Norwegian School Fruit Program for free during the school year 2001/2002 [26]. The free subscription program started in October 2001 and lasted throughout the school year (i.e. until June 2002). The pupils in the intervention schools received a piece of fruit or a carrot per day for free each school day for one year, usually in connection with their lunch meal [26]. Apples, pears, bananas, oranges, clementines, kiwis, carrots and nectarines were the most frequently FV given. The remaining 29 schools served as control schools [27]. A more detailed description of the study design has previously been published [28]. The free fruit was given in addition to the normal Norwegian lunch, typically consisting of sandwiches brought from home. There was no focus on decrease of other foods items within the free fruit program. However, we have previously observed a decrease in consumption of unhealthy snacks following the intervention participants compared to the controls, both in the current cohort [29], and also when evaluating the later nationwide implementation of free school fruit lasting from 2007 to 2014 [30].

Some pupils also received an educational program during the intervention year (2001/2002), however a previous publication showed no effect on fruit and vegetable intake of this educational program [31,32]. Therefore, in the current study we only distinguish between those who received free fruit and those who did not.

### Procedure

In September 2001, 6^th^ and 7^th^ graders (age 10–12) completed the baseline survey (before the fruit program started) [26]. Follow-up surveys were conducted in May 2002, May 2003, May 2005 and September 2009 [27], and data collection for a fifth follow-up survey started in January 2016 and ended in November 2016. In the present study, the data collections from 2001, 2005 and 2016 are used. Data from the 2002—and 2003 surveys are not included in this study because the surveys did not include questions regarding weight status. In addition, the 2003 survey only included half of the study sample [28]. Data from the 2009 survey is not included in the present study due to the low participation rate and the fact that results from this follow-up survey have been presented earlier [22,27].

The surveys conducted in the period between 2001 and 2005 were completed by the pupils in the classroom in the presence of a trained project worker [33]. One school lesson (45 min) was used to complete the questionnaires. In 2016, eligible participants were contacted through Facebook or by phone, and were asked to conduct the survey for the fifth follow-up. This strategy was chosen as at that time the participants did not attend schools anymore and Facebook seemed a promising tool to find people.

The FVMM study was conducted according to the guidelines laid down in the Declaration of Helsinki. Ethical approval and research clearance were obtained from The National Committees for Research Ethics in Norway (REK) (file number S-01076) and from The Norwegian Social Science Data Services (NSD) (file number 12395). Written informed consent was obtained from all subjects, i.e. the participants themselves and their parents.

### Study sample

Of 2287 eligible schoolchildren, a total of 1950 pupils (85%) completed the survey at baseline. Fifty-nine (3%) children–parents refused to participate, one class (27 children, 1%) was not able to carry out the survey, and 251 (11%) children did not attend this specific school lesson and were not re-contacted for the baseline survey [26]. Those that initially refused to participate (n = 59), and those who resigned throughout the follow-ups (n = 179) were not contacted for the 2016 survey, thus 2049 were eligible for the 2016 survey, and a total of 1081 participants (53%) did participate. The average age in 2016 was 26.5 years. Of the study sample, a total of 190 participants (17.6% of responders, 9.3% of eligible sample) reported to have one or more children. In total, 270 children were included.

### Instruments

Self-reported perceived weight status was assessed by the following question at all surveys: “Are you on (slimming) diet”, with the following response alternatives: “No, my weight is OK”, “No, but I should be on a (slimming) diets”, and “yes”. The two last alternatives were seen to indicate that the responders perceive themselves as too heavy/overweight. A new variable was calculated by combining these two to: 1 = I am too heavy, 0 = my weight is OK [22].

Self-reported height and weight were included in the 2005 and 2016 surveys, but not in the baseline survey conducted in 2001. The reason for not including height and weight in the original survey, was that the main aim of the original study was to evaluate the intervention on fruit and vegetable intake and its behavioral determinants and not changes in BMI [22]. In the present study, BMI was calculated based on self-reported height and weight. For the 2005 survey, age and sex-specific cut off points according to the International Obesity Task Force (IOTF) definitions [34] were used to categorize the adolescents for being overweight or obese (≥25 kg/m^2^). Furthermore, World Health Organization’s (WHO) definition of adult overweight and obesity (>18 years) was used for the classification of the participants in the 2009 and 2016 survey (≥25 kg/m^2^) [35]. For the present analyses, the variable was dichotomized into overweight/obese (≥25 kg/m^2^) vs. not overweight/obese (<25 kg/m^2^).

In the 2016 survey, questions regarding the participants’ children were included. The first question was formulated “Do you have children”, with the following alternatives: “No” and “Yes”. In total, it was possible to register four children. If the responder answered “Yes”, two new questions were asked: “In what year is it born?” and “What was the birth weight of the new-born (in gram)?”. Birth weight variable was trichotomized into three categories: high birth weight (HBW) (≥4000 gram), low birth weight (LBW) (<2500 gram), and normal birth weight (2500–3999 gram). In the regression analysis, this variable was dichotomized into ‘normal birth weight’ vs. ‘low birth weight’/’high birth weight’ to assess the odds of not being in recommended weight category.

In the surveys, the participants reported their own gender and age. In the 2016 survey, the participants recorded their own educational level as an indicator of socioeconomic status. The educational level variables were dichotomized into lower education (no university or college) or higher education (university or college).

### Statistical analyses

Descriptive statistics were used to characterize the current study samples at different time points. Comparisons were made between those participating in the intervention group and the control group. For these comparisons χ^2^ statistics were used for the categorical variables. Independent Sample t-test was used for the continuous variables. All the continuous variables used in the crude analyses were considered as normally distributed.

Logistic (multilevel) regression analyses was used to assess the association of the intervention condition with weight status (0 = normal weight, 1 = overweight) and with birth weight (0 = normal birth weight, 1 = low or high birth weight). Assumptions regarding multicollinearity were checked by calculating correlation coefficients between all co-variates and these did not indicate multicollinearity problems. Furthermore, the assumption of independence of errors was violated as participants provided more than one assessment. This violation was solved by using multilevel analyses that takes into account the dependency between observations within the same person [36].

Gender and the participant’s education level were included in these analyses as potential confounders. For the analyses with weight status of parents as an outcome, two levels were defined: (1) participants and (2) schools. However, based on changes in the -2 Log Likelihood (-2LL) relative to the changes in degrees of freedom (df), adding school as a level did not significantly improve the model (Δ -2LL = 0.10, Δ df = 1, p = 0.103). Aiming for the most parsimonious model the school level was therefore omitted.

For the analysis with birth weight as an outcome, this model was defined with three levels (1) children, (2), participants (parents) and (3) schools. However, adding school as a level in the model did not improve the model compared to a model not adjusting for the nested design (Δ -2LL = 0, Δ df = 1, p = 1.000). Therefore, we decided not to adjust for the school level and tested the model that was adjusted for the parent level only, which improved the model slightly (-2LL = -139.88, df = 5 vs. -2LL = -138.06, df = 6, Δ = 1.82, df = 1). Despite the non-significant improvement of the model after adding the parental level, we decided to keep this for theoretical reasons. Children from the same parents resemble each other more regarding their birth weight than they resemble other children. Therefore, the model was re-defined with only two levels, (1) children and (2) participants (parents). Besides the study participants’ intervention condition (intervention vs. control), the following variables were included in the models as potential confounders: their gender, their educational level and for the model on birthweight also the participants (i.e. parents of the newborns) BMI (dichotomized into overweight vs. not overweight).

All the descriptive analyses were conducted using IBM SPSS Statistics 24.0. R software, version 3.2.2, was used to run the multilevel binary logistic regression models (lme4 package). All tests were two-sided with p-values <0.05 considered statistically significant.

## Results

In the present study (n = 1081), 322 participants were in the intervention group and 759 in the control group. No significant differences were observed between those groups regarding gender. There was a significantly higher prevalence of participants with high educational level in the intervention group compared to the control group (p = 0.035) (Table 1). The total number of children in the present study was 270; 67 of them belonged to a parent from the intervention group, 203 children to a parent from the control group. The first offspring was born in 2000, but most participants had their first child after 2011 (75% of the first children was born in the years from 2012 till 2016). No significant differences were observed between those groups regarding gender, educational level or weight status of their parent (Table 1). Data for characterization of participants at different time points is provided in S1 Table.

**Table 1:** Characteristics of the present participants (n = 1081) including the offspring of the participants (n = 270).

	Intervention group n(%)	Control group n(%)	p-value*
**Study participants**	322(29.8)	759(70.2)	
Gender			
Male	148(47.0)	328(44.4)	*0*.*478*
Female	167(53.0)	411(55.6)	
Age in 2016 (mean (SD))	26.5 (0.53)	26.5 (0.51)	*0*.*815*^ǂ^
Education level			
Low	90(28.7)	262(35.6)	*0*.*035*
High	224(71.3)	474(64.4)	
**Offspring of the participants**			
Number of children	67(24.8)	203(75.2)	
Parents’ gender ^1^			
Male	21(31.3)	63(31.0)	*1*.*000*
Female	46(68.7)	140(69.0)	
Parents’ education ^1^			
Low	33(49.3)	128(63.4)	*0*.*058*
High	34(50.7)	74(36.6)	
Parents’ weight^1^			
Overweight/obese (BMI≥25)	30(46.9)	92(46.0)	*1*.*000*
Not overweight/obese (BMI<25)	34(53.1)	108(54.0)	

*based on χ2 (Yates’s continuity correction)

^ǂ^ based on ANOVA; Overweight: BMI ≥25

Lower education level: no university or college; High education level: university or college

^1^ Parent of the offspring refers to the participant in the FVMM study

The prevalence of overweight increased from 11% in 2005 to 34% in 2016 for the intervention group. In the control group, this prevalence increased from 11% in 2005 to 36% in 2016 (Table 2). These differences between the intervention and the control group were not statistically significant. The prevalence of normal birth weight was 80% among children with parents in the intervention group, compared to 72% among children with parents in the control group (p = 0.260). Furthermore, the prevalence of LBW was 3% in the intervention group, compared to 8% in the control group (p = 0.299), and the prevalence of HBW was 17% in the intervention group, and 20% in the control group (p = 0.678) (Table 2). Data on birth weight of offspring and parental weight status 14 years after participation in the intervention group or the control group is provided in S2 Table.

**Table 2:** Observed means (SD) and prevalence at all time points for the participants (n = 1081) and for the offspring (n = 270).

Year	2001			2005			2016		
	Mean (SD) or %			Mean (SD) or %			Mean (SD) or %		
	Intervention	Control	*p-value*	Intervention	Control	*p-value*	Intervention	Control	*p-value*
Weight (kg)^a^	No data			59.6 (11.0)	60.0 (12.1)	*0*.667*	75.2 (16.3)	74.1 (15.4)	*0*.300*
Height (cm)^a^	No data			169.6 (8.5)	170.2 (9.0)	*0*.432*	174.5 (9.6)	174.2 (9.6)	*0*.614*
BMI (kg/m^2^)^a^	No data			20.6 (3.2)	20.6 (3.6)	*0*.*937*	24.5 (4.2)	24.3 (3.9)	*0*.*405*
Overweight (%)^b^	No data			11.0	10.8	*1*.000**	33.7	35.7	*0*.571**
Stating themselves to be too heavy (%)^b^	22.5	23.7	*0*.749**	27.4	30.4	*0*.428**	35.3	35.2	*1*.000**
*Offspring*									
Birth weight (gram)^a^							3523 (554)	3461 (688)	*0*.515*
High birth weight (%)^b^							17	20	*0*.678**
Normal birth weight (%)^b^							80	72	*0*.260**
Low birth weight (%)^b^							3	8	*0*.299**

^a^ The data are presented as means (SD)

^b^ The data are presented in %; BMI: <18.5 underweight, 18.5–24.9 normal weight, ≥25 overweight

*based on independent t-test for continuous data

**based on χ^2^ (Yates’s continuity correction); High birth weight: ≥4000 gram, Normal birth weight: 2500–3999 gram, Low birth weight: <2500 gram

Multilevel logistic regression analysis (Table 3), showed that the odds of being overweight was 0.91 (95% CI: 0.69, 1.21) for the intervention group compared to the control group (Table 3, model 1). After adjusting for gender and educational level, the odds of being overweight was 0.93 (95% CI: 0.70, 1.24) for the intervention group, compared to the control group (Table 3, model 3).

**Table 3:** Odds Ratios (OR) and 95% Confidence Intervals (CI) for being overweight in 2016 for the participants or having children with low or high birth weight for the intervention group compared to the control group.

	Model I		Model II			Model III		
	OR	95%CI	OR	95%CI		OR		95%CI
Being overweight (participant)	(n = 1034)		(n = 1034)			(n = 1033)		
	0.91	(0.69;1.21)	0.89	(0.67;1.18)		0.93		(0.70;1.24)
Having a child with low or high birth weight	(n = 257)		(n = 256)			(n = 254)		
	0.55	(0.22;1.38)	0.55*	(0.22;1.38)		0.52**		(0.21;1.30)

Model I–crude model, Model II–Model I + gender, Model III–Model II + educational level

* additionally adjusted for educational level of the participant

** additionally adjusted for overweight status (BMI>25) of the participant (parents).

The odds of having a child with high or low birth weight was 0.55 (95% CI: 0.22, 1.38) in the intervention group compared to the control group (Table 3, model 1). Further, the odds of having a child with high or low birth weight was 0.52 (95% CI: 0.21, 1.30) for the intervention group compared to the control group after adjusting for gender, educational level and BMI (Table 3, model 3).

## Discussion

Findings from the present study showed that providing free school fruit for one year in elementary school had no statistically significant effect on weight status of the participants 14 years later. Previously, we have reported a reduced prevalence of overweight in the intervention group compared to the control group after seven-year follow-up, but the effect was not significant after adjusting for gender and parental education [22]. Lack of statistically significant results in the crude model presented in the present study, may be partly explained by the long follow-up period and/or the much larger (and therefore more representative) sample size compared to the seven-year follow-up study.

Previously published systematic review studies have reported a lack of evidence showing an association between fruit and vegetable intake and weight status and that many studies investigating this relationship has been hampered by low study quality [37,38]. However, a ten-year observational follow-up study showed a reduced weight gain among healthy adults with a high intake of FV at the beginning of the study period [39], and a comprehensive observational study among women showed that a diet rich in fruit and vegetables was associated with less weight gain over a 14-year period [40].

A few other intervention studies promoting healthy eating habits among children have shown significant reductions in BMI two and three years after the intervention period [41–43]. Based on these studies and other studies showing a dose-response association between pre-pregnancy BMI and offsprings`birth weight (5,6,8), we hypothesized that a one-year intervention promoting fruit and vegetable intake could affect weight status of participants on the long term, and possibly also affect birth weight of their children. Results from the present study, however, showed no statistically significant differences in birth weight status among participants offspring in the intervention group and those participating in the control group.

As few previously published FV intervention studies have evaluated the long-term effect on weight status [44], a major strength of the present study was the extended follow-up period and the effect evaluation on both participants and their children. Another important strength of the FVMM study was the partly randomized intervention design, and that participants were enrolled prior to pregnancy. It is well-known, however, that results may be biased due to differences in background characteristics between the study sample and those lost to follow-up [45]. Among those participating in the last follow-up study (2016) a significantly higher educational level was shown in the intervention group compared to the control group. No other differences in characteristics were observed between the groups.

The present study has also several limitations that should be considered. We did not collect baseline measures of weight and height, and the subsequent measures of participants´ weight and height and birth weight of their children were self-reported. Based on the fact that overweight or obese peoples might tend to understate their weight [46], it is possible that errors have occurred in the process of grouping the BMI of the participants. In addition to the weight and height of the participants, the birth weight of their children was self-reported. The gestational age and prevalence of preterm birth was unknown as well. Furthermore, the intervention schools were only randomized within one of the two participating counties [29]. Hence, the FVMM intervention study was not perfectly randomized, and the control group also included nine schools that from the school year 2001/2002 participated in the Norwegian school fruit subscription program (a parental payment version). Some of the participants also received an FV educational program during the intervention year (2001/2002), but we have previously confirmed that this part of the program did not affect fruit and vegetable intake [32]. Finally, the present study was limited by a low number of participants´ children included in the study, which most likely resulted in low statistical power and reduced possibility to detect possible intervention effects on birth weight of the children. As the present study included only a few children with low birth weight in particular, but also a relatively few children with high birth weight, these categories were merged to compare normal birth weight with non-normal birth weight. Unfortunately, this merging of categories caused loss of relevant information. Thus, future follow-up studies should include a higher number of participants which will most likely result in a sufficient number of participants in each weight category and thereby assuring an adequate power to detect statistically significant associations.

## Conclusion

Results from the present study did not show an intervention effect on participants´ weight status 14 years later or on the birth weight of their children. In order to conclude whether free school fruit may have a protective effect on the risk of developing overweight or obesity or on birth outcome, longer term follow-up studies with larger samples are needed. Future studies should also explore whether provision of free school fruit for more than one year may positively effect weight status in adulthood.

## Supporting information

S1 Table Data describing characteristics of the participants at different time points. (SAV)

S2 Table Data on birth weight of offspring and parental weight status 14 years after participation in the intervention group or the control group of the free school fruit intervention. (SAV)

## Abbreviations

-2LL: -2 Log Likelihood
BMI: Body Mass Index
CI: Confidence Interval
df: degrees of freedom
FFQ: Food Frequency Questionnaires
FV: Fruit and Vegetables
FVMM: Fruits and Vegetables Make the Marks
IOTF: International Obesity Task Force
OR: Odds Ratio
SES: Socioeconomic Status
WHO: World Health Organization
